# Pan-Genome Analysis of the Fructokinase Gene Family Reveals a Light-Regulated SiPhyC–SiFRK4 Module Controlling Carbon Partitioning in Foxtail Millet

**DOI:** 10.3390/plants15060907

**Published:** 2026-03-15

**Authors:** Lu He, Juan Zhao, Guangxin Wang, Ling Yuan, Xingchun Wang, Zhirong Yang

**Affiliations:** 1 Houji Laboratory in Shanxi Province, College of Agriculture, Shanxi Agricultural University, Taigu, Jinzhong 030801, China; luhe505@foxmail.com (L.H.);; 2 Maize Research Institute, Shanxi Agricultural University, Xinzhou 034000, China; 3 Department of Basic Sciences, Shanxi Agricultural University, Taigu, Jinzhong 030801, China; zhaojuan121768@163.com (J.Z.);; 4 Department of Plant and Soil Sciences, Kentucky Tobacco Research and Development Center, University of Kentucky, Lexington, KY 40546, USA; 5 College of Life Sciences, Shanxi Agricultural University, Taigu, Jinzhong 030801, China

**Keywords:** foxtail millet, fructokinase, fructose, pan-genome, *SiFRK4*, *SiPhyC*

## Abstract

Fructokinase (FRK) initiates fructose phosphorylation, channeling carbon into central metabolic pathways, yet its functional diversity and regulatory networks in C_4_ cereals remain poorly understood. Here, we performed a comprehensive pan-genome analysis of the *FRK* gene family in foxtail millet (*Setaria italica*), identifying 697 *SiFRKs* across 110 accessions and revealing extensive presence–absence variation shaped by evolution and domestication. Among nine characterized members in the reference genome, *SiFRK4* exhibited broad and high expression, a diurnal rhythm, and substantial natural variation. Biochemical assays confirmed its fructokinase activity in vitro. We discovered a novel physical interaction between SiFRK4 and the key photoreceptor Phytochrome C (SiPhyC), which co-localized in the cytoplasm. Functional analysis of *SiPhyC* mutants demonstrated that loss of *SiPhyC* disrupted carbohydrate homeostasis, elevating fructose while depleting sucrose and starch. Our findings reveal a physical and genetic link between the light-signaling component SiPhyC and the metabolic enzyme SiFRK4, suggesting their interaction influences carbon partitioning. This study provides foundational insights into the sugar metabolism network of a resilient C_4_ model crop and identifies potential targets for metabolic engineering and breeding.

## 1. Introduction

FRK is a key enzyme in fructose metabolism with dual subcellular localization and diverse functions in plants. As a member of the PfkB family, FRK proteins contain highly conserved PfkB domains, ATP-binding sites, and substrate recognition regions [1]. Functionally, plant FRKs fall into two main categories: cytosolic FRKs phosphorylate fructose to fructose-6-phosphate, feeding directly into glycolysis, while plastid-localized FRKs channel fructose into the Calvin cycle and other plastid pathways such as amino acid metabolism [2]. Most plant species possess a single plastid-localized FRK, but maintain multiple cytosolic isoforms [2]. Gene family analyses reveal considerable species-specific variation in *FRK* copy number. For example, the dicot model *Arabidopsis* contains seven *FRK* genes [3], whereas monocot plants such as rice, maize, sorghum, and foxtail millet harbor six, six, seven, and nine members, respectively [2].

To date, multiple *FRK* genes have been cloned and characterized in species such as *Arabidopsis*, tomato, and rice, underscoring their functional versatility [3,4,5]. *FRKs* participate in various aspects of plant metabolism. In apple, *MdFRK2* contributes to sucrose and sorbitol metabolism in leaves and promotes cellulose synthesis by enhancing fructose metabolism [6,7]. Cucumber *FRKs* are involved in chlorophyll biosynthesis [8], while an *Arabidopsis frk* mutant shows reduced seed oil content, suggesting a link with lipid metabolism [9]. In rice, *OsFRK3* influences grain filling by modulating starch accumulation [10]. Beyond metabolism, *FRKs* also regulate plant growth and development. Tomato *Frk1* is essential for phloem fiber development and floral induction at the shoot apical meristem; antisense suppression of *Frk1* delays flowering, whereas inhibition of *Frk2* impairs stem and root growth and reduces flower, fruit, and seed numbers [11]. *SlFRK3* regulates xylem development and affects water transport [12]. In *Arabidopsis*, the *atfrk3*/*atfrk1* double mutant exhibited delayed flowering under short-day conditions, and AtFRK6 interacted with TWIN SISTER OF FT (TSF), a homolog of the florigen FT, to fine-tune the short-day response [13]. Moreover, FRKs act as carbon flux regulators during stress adaption. Rice OsFRK1 and OsFRK2 are implicated in hypoxia tolerance [14], maize ZmFRK2 responds to salt stress [15], and apple MdFRK2 affects drought tolerance via the abscisic acid pathway [16]. Despite these advances, a systematic analysis of the *FRK* gene family in C_4_ cereals remains lacking, and their evolutionary patterns, functional characteristics, and regulatory networks are not well understood.

Foxtail millet, a C_4_ cereal crop originating in China, is renowned for its drought tolerance, adaptation to nutrient-poor soils, and broad environmental resilience. It serves as a key crop for dryland agro-ecology and a strategic reserve for coping with extreme climates. With advantages such as self-pollination, diploid genome, and compact genome size, foxtail millet has emerged as an excellent model for studying C_4_ photosynthesis and stress adaptation [17,18,19]. Recent advances in foxtail millet genomics and bioinformatics have enabled genome-wide identification of numerous gene families, including *MADS-box* and *bHLH* genes [20,21,22,23], and the discovery of genes underlying yield, quality, and stress-resistance traits [24,25,26,27,28,29]. Research on sugar metabolismhas employed multi-omics approaches to identify key enzymes involved in starch synthesis and sugar degradation—e.g., ADP-glucose phosphorylase, starch synthase, starch-branching enzyme, and cell-wall invertase—and to delineate their expression dynamics and regulatory networks during grain filling and quality formation [23,30]. Sugar metabolism also plays a critical role in foxtail millet’s adaptation to abiotic stresses such as drought, salinity, alkalinity, and low temperature, during which genes encoding sucrose synthase and sucrose-phosphate synthase show marked expression changes that promote stress acclimation [31,32,33,34]. Nevertheless, the overall molecular circuitry governing sugar metabolism in foxtail millet remains unclear, particularly with respect to the contributions of individual enzyme families, signaling pathways, and their interactions. Hexokinase and fructokinase are two key kinases that initiate hexose phosphorylation, providing entry points into downstream metabolic pathways. While the hexokinase family in foxtail millet has been studied and linked to stress responses and yield traits [35], the physiological functions, regulatory mechanisms, and agronomic impacts of fructokinase have not been systematically investigated.

Here, we performed a comprehensive analysis of the *FRK* gene family in foxtail millet using pan-genomic data from 110 accessions, identifying 697 *SiFRK* members through bioinformatics approaches. We focused on the highly expressed *SiFRK4* for biochemical and functional validation. This work lays a molecular foundation for elucidating the sugar-metabolism network in foxtail millet and provides novel genetic targets for crop improvement.

## 2. Results

### 2.1. Pan-Genome Identification of the SiFRK Gene Family

**Figure 1:**
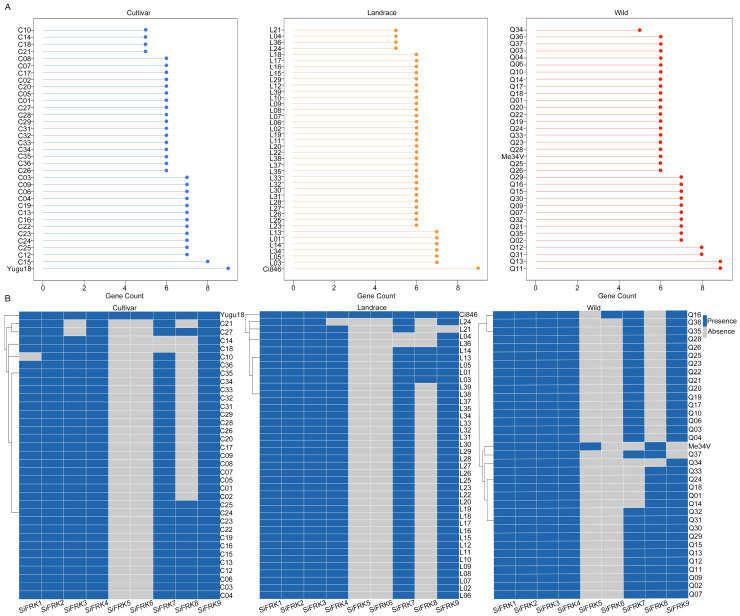
Identification of *SiFRK* genes in 110 foxtail millet accessions. (**A**) Statistical analysis of *SiFRK* genes across foxtail millet accessions (**B**) PAV analysis of *FRK* family members in each foxtail millet accession. Blue indicates the presence of the gene, while gray indicates its absence.

To systematically assess the genetic diversity and evolutionary landscape of the *SiFRK* gene family in foxtail millet, we performed a pan-genome analysis across 110 accessions, including 35 cultivars, 40 landraces, and 35 wild relatives. In total, we identified 697 *SiFRK* genes, with 223 present in cultivars, 245 in landraces, and 229 in wild relatives (Figure 1A). The lists of 697 *SiFRK* genes and their coding sequence (CDS) sequences are provided in Supplementary Data S1 and S2. The number of *SiFRK* genes per accession ranged from 5 to 9. Nine members were found in Yugu18, Ci846, Q11 and Q13, whereas eight genes were detected in C15, Q12, and Q31. Most accessions (66) harbored six *SiFRK* genes, while 28 possessed seven, and nine carried only five.

To further evaluate the presence–absence variation (PAV), we analyzed the distribution patterns of individual *SiFRK* members across all accessions (Figure 1B). *SiFRK2* was present in every accession, indicating strong evolutionary conservation. *SiFRK1* and *SiFRK3* were mainly detected in wild relatives and landraces, whereas *SiFRK4* was broadly distributed across wild relatives and cultivars. In contrast, *SiFRK9* was predominantly found in cultivars. The remaining members—*SiFRK5*, *SiFRK6*, *SiFRK7*, and *SiFRK8*—displayed notable absence patterns. Specifically, *SiFRK5* and *SiFRK6* were each present in only three accessions, and the *SiFRK8* gene was absent in 65.5% of the accessions. These patterns suggest that certain *SiFRK* members may have experienced selective pressure during ecotype differentiation or breeding, leading to recurrent loss throughout evolution.

**Figure 2:**
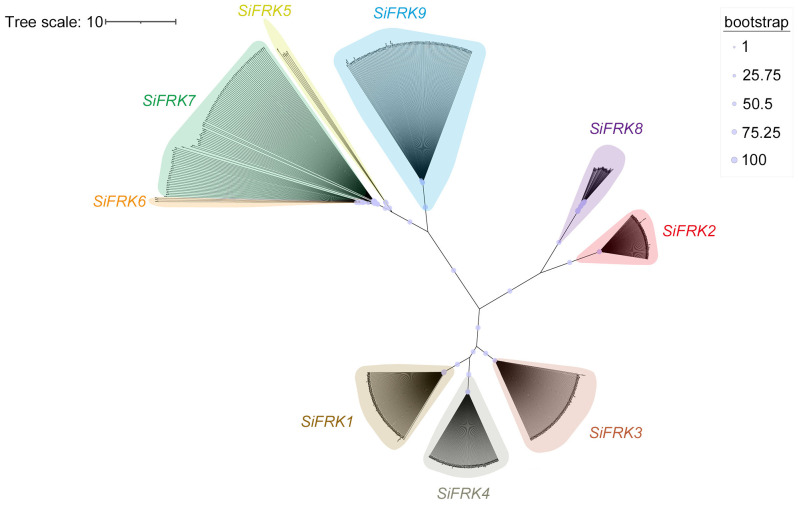
Phylogenetic tree analysis of 697 SiFRK proteins in 110 foxtail millet accessions. Bootstrap values are represented by the size of the circles at nodes. Larger circles indicate higher support.

To elucidate the evolutionary relationships within the *SiFRK* gene family, we constructed a phylogenetic tree using the maximum likelihood method based on full-length SiFRK protein sequences from Yugu1 and the 110 foxtail millet accessions (Figure 2). The SiFRK members were divided into four distinct clades: SiFRK1, SiFRK3, and SiFRK4 clustered together; SiFRK2 and SiFRK8 showed closer affinity; SiFRK5, SiFRK6, and SiFRK7 formed an independent clade; and SiFRK9 constituted a separate clade, suggesting it might have unique biological functions.

### 2.2. Bioinformatic Characterization of the SiFRK Gene Family in Foxtail Millet

To systematically characterize the *SiFRK* genes, we conducted a comprehensive bioinformatic analysis of the nine *SiFRK* members identified in the high-quality telomere-to-telomere (T2T) genome of the reference cultivar Yugu1 (Table 1). The proteins encoded by these genes ranged from 323 to 581 amino acids (34.65 to 64.90 kDa). Among them, proteins in the SiFRK2/8 clade were longer, resulting in higher molecular masses compared to members of other clades. Their predicted isoelectric points (PI) varied between 4.85 and 7.56, while grand average of hydropathicity (GRAVY) values spanned from −0.630 to 0.154, indicating that most SiFRK proteins were hydrophobic.

**Figure 3:**
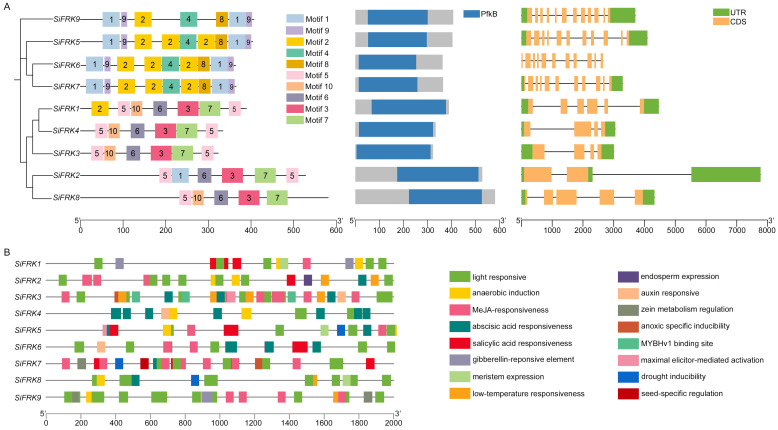
Bioinformatic characterization of the *SiFRK* gene family in foxtail millet. (**A**) Conserved protein motifs, functional domains and exon–intron structures of the nine *SiFRK* genes. (**B**) Distribution of cis-regulatory elements in the promoter regions.

Analysis of gene structures and conserved protein motifs revealed further insights into family organization (Figure 3A). The number of exons in *SiFRK* genes varied from 3 to 12, corresponding to 2 to 11 introns. Members of the SiFRK5/6/7 clade, along with the closely related SiFRK9, possessed 11–12 exons, whereas those in other clades contained only 3–6 exons. Motif composition was largely conserved within phylogenetic clades. Members of the SiFRK5/6/7 clade and the closely related SiFRK9 shared a core set of motifs (Motifs 1, 2, 4, 8, and 9), whereas other members primarily contained Motifs 3, 5, 6, and 7—suggesting these motifs might underlie specific functional or catalytic properties. All nine proteins contained the characteristic fructokinase PfkB domain, confirming their annotation as functional FRK homologs. These results suggested distinct structural characteristics among the four clades, indicating potential functional divergence.

To investigate transcriptional regulation, we analyzed cis-acting elements within the 2-kb promoter regions upstream of each *SiFRK* transcription start site (Figure 3B). Promoters contained diverse regulatory elements associated with light responsiveness (e.g., G-box, GT1-motif, Sp1), hormone signaling (e.g., ABRE, CGTCA-motif, TGACG-motif), stress responses (e.g., LTR, MBS, TC-rich repeats), and tissue-specific expression (e.g., RY-element, CAT-box, GCN4 motif). Light-responsive elements were ubiquitous, suggesting photoregulation might be a common feature of *SiFRK* expression. Notably, the promoter of *SiFRK4* was enriched in abscisic acid-responsive elements (ABREs), implicating this gene in ABA-mediated stress and metabolic signaling. In addition, the presence of a seed-specific RY-element in the *SiFRK7* promoter pointed to a potential role in seed development or germination.

To investigate structural variation within the *SiFRK* gene family and its potential contribution to functional diversity, we performed a comparative analysis of gene structures (exon–intron organization) and conserved motif compositions for nine *SiFRK* genes across 110 accessions from our pan-genome dataset (Figures S1–S9). This analysis revealed distinct evolutionary trajectories among family members. *SiFRK4* exhibited a highly conserved structure across all 109 accessions in which it was present, consistently comprising four exons, with only length polymorphisms detected in the first exon and intronic regions. In contrast, *SiFRK7*, *SiFRK8*, and *SiFRK9* displayed pronounced structural diversity, each showing multiple distinct exon–intron configurations across accessions. The remaining *SiFRK* members maintained highly consistent structures, with variations observed only rarely. These contrasting patterns suggest differential evolutionary constraints acting upon individual gene family members, likely reflecting their specialized functional roles or regulatory mechanisms. Analysis of conserved motifs further corroborated this functional divergence. Within each gene, the core motif backbone—typically corresponding to the protein kinase catalytic domain—remained largely intact across different accessions. For instance, SiFRK3 consistently contained Motifs 1, 2, 3, 4, and 5 in all accessions where the gene was present. Notably, variations in motif composition were observed and correlated with structural polymorphisms. SiFRK7, for example, exhibited multiple motif composition types, indicating that structural polymorphisms at the DNA level can directly impact the integrity and arrangement of functional protein domains. These pan-genome-scale results capture both the conserved core functions and the structural diversity within the *SiFRK* family, providing deeper insight into their functional evolution.

### 2.3. Expression Profiling of SiFRK Genes Reveals Stage- and Tissue-Specific Patterns

**Figure 4:**
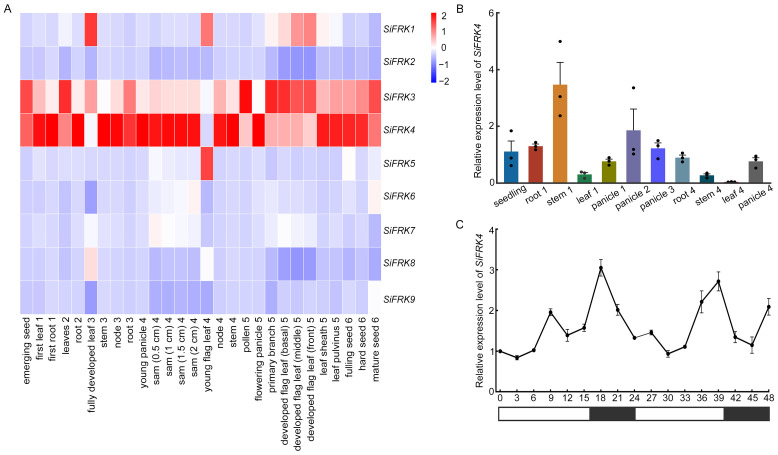
Expression patterns of *SiFRK* genes in foxtail millet. (**A**) Transcript levels of *SiFRK* genes at different developmental stages based on public transcriptome data of cultivar Yugu1. Expression values (Fragments per kilobase of transcript per million mapped reads, FPKM) are represented in the heatmap, with color intensity ranging from blue (low expression) to red (high expression). Developmental stages: 1, seedling; 2, three-leaf; 3, shooting; 4, booting; 5, flowering; 6, mature. (**B**) Tissue-specific expression of *SiFRK4* in cultivar JG21 across developmental stages as measured by RT–qPCR. Stages analyzed: 1, booting; 2, heading; 3, flowering; 4, grain filling. Data are presented as mean ± S.D. of three biological replicates (*n* = 3). (**C**) Diurnal expression profile of *SiFRK4*. Light and dark periods are indicated by white and black bars, respectively. Data are presented as mean ± S.D. (*n* = 3).

To elucidate the biological functions of the *SiFRK* gene family in foxtail millet, we systematically analyzed the expression profiles of its nine members across various tissues and developmental stages. Based on public transcriptome data from the Yugu1 variety, an expression heatmap revealed distinct spatiotemporal patterns among the *SiFRK* genes (Figure 4A). *SiFRK1* exhibited high expression in leaves at multiple stages after shooting. *SiFRK3* was strongly expressed in several tissues–excluding panicles–during flowering and maturation, with additional elevated levels in germinated seeds, three-leaf-stage leaves, as well as roots and leaves at the shooting stage. *SiFRK4* showed consistently high expression across most tissues at different developmental phases, except for lower expression in leaves during the shooting and booting stages. *SiFRK5* was preferentially expressed in flag leaves at the booting stage, while the remaining genes displayed relatively low expression throughout development.

We further focused on the expression pattern of *SiFRK4* in the JG21 variety (Figure 4B). *SiFRK4* expression was highest in stems at the booting stage, followed by panicle at heading stage, whereas expression in leaves remained low during both booting and filling stages. During panicle development, *SiFRK4* transcript levels increased initially, peaked at heading, and subsequently declined. These results suggest that *SiFRK4* likely plays a role in the heading process of foxtail millet. Further analysis of the circadian expression of *SiFRK4* gene revealed that expression peak occurred around the end of the light period (Figure 4C), suggesting that its expression was regulated by the circadian clock.

### 2.4. Natural Variations of the SiFRK Genes in Foxtail Millet

**Figure 5:**
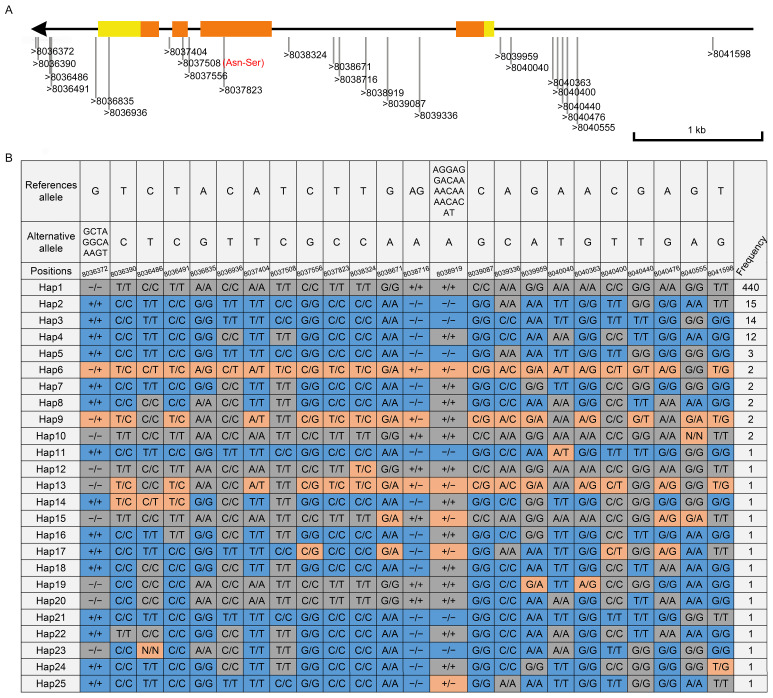
Natural variations in the *SiFRK4* gene. (**A**) Schematic representation of genetic variations in *SiFRK4*. Exons are depicted as filled boxes, with introns and promoter sequences represented by lines. UTRs are indicated by yellow filled boxes, while coding sequences are shown in orange filled boxes. The direction of transcription is indicated by arrows. Red letters denote non-synonymous mutations. (**B**) Haplotypes of the *SiFRK4* gene identified in 509 germplasm accessions. The *SiFRK4* sequences from 508 accessions were compared with that of the reference sequence Yugu1 (Hap1). A total of 25 haplotypes were identified among these accessions. Blue boxes represent sequences identical to the Yugu1 reference, gray boxes indicate variant sites, and orange boxes denote heterozygous sites. The number of accessions corresponding to each haplotype is shown on the right.

Natural variation plays a critical role in revealing gene function, elucidating evolutionary processes, and advancing crop breeding. To further explore the evolution of the *SiFRK* gene family and facilitate the use of diverse natural germplasm, we performed a comprehensive haplotype analysis based on resequencing data from 509 foxtail millet accessions, including modern cultivars, traditional landraces, and wild relatives of *Setaria viridis*. Our analysis revealed abundant genetic variation in six of the *SiFRK* genes, whereas no variation was detected in *SiFRK2*, *SiFRK7*, or *SiFRK8* (Figure 5 and Supplementary Data S3). Among the variable genes, *SiFRK5* exhibited the highest number of polymorphic sites (36), with two nonsynonymous mutations resulting in amino acid changes (Ala to Ser at position 228 and Val to Leu at position 302). In contrast, *SiFRK9* displayed the fewest, with only three polymorphic sites. Notably, the *SiFRK4* gene also displayed substantial polymorphism, with a total of 24 variation sites identified, including three InDels and 21 single nucleotide polymorphisms (SNPs). Among these variants, 8 were located in the promoter region, 1 in the 3′ untranslated region (UTR), 2 in exonic regions, 8 within introns, and the remaining 5 in the downstream region. Of the two exonic SNPs, one was a non-synonymous mutation leading to Asn to Ser amino acid changes at position 264 and another was a synonymous variant. Collectively, these variations defined 25 distinct haplotypes, underscoring a rich reservoir of natural diversity for this gene within the surveyed population (Supplementary Data S3).

### 2.5. SiFRK4 Exhibits Fructokinase Activity In Vitro

**Figure 6:**
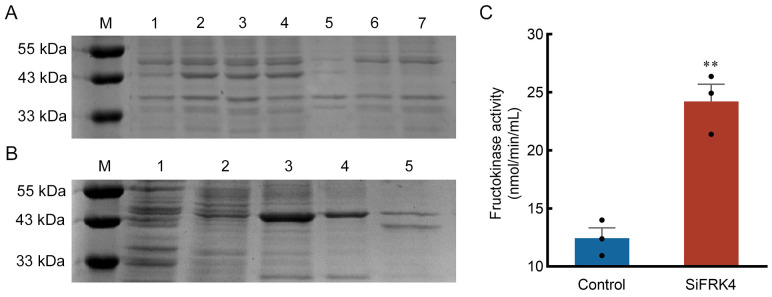
In vitro fructokinase activity of SiFRK. (**A**) SDS-PAGE analysis of SiFRK expression in *E. coli* BL21. (M: 180 kDa Marker. Lane 1: pET28a(+)-SiFRK without Isopropyl-β-D-thiogalactopyranoside (IPTG) induction. Lane 2: pET28a(+)-SiFRK induced with 1 mM IPTG. Lane 3: total lysate of IPTG-induced pET28a(+)-SiFRK cells. Lane 4: soluble fraction of the lysate. Lane 5: insoluble fraction of the lysate. Lane 6: pET28a(+) empty vector without IPTG. Lane 7: pET28a(+) empty vector induced with 1 mM IPTG. (**B**) SDS-PAGE electropherograms of SiFRK protein purified by imidazole elution at different concentrations. M: 180 kDa Marker. Lane 1: flow-through fraction. Lane 2−5, proteins eluted with 10, 25, 50 and 100 mM imidazole, respectively. (**C**) In vitro fructokinase activity of purified SiFRK. The lysate of BL21 cells harboring the empty pET28a(+) vector was used as a negative control. The scatter points represent the measured enzyme activity values. Data are presented as mean ± S.D. from three independent experiments. Statistical significance was determined by one-way analysis of variance (ANOVA) followed by LSD’s significant difference test. Asterisks denote a statistically significant difference (** *p* < 0.01).

To characterize the biochemical activity of SiFRK4, its CDS was cloned into the pET28a(+) expression vector and heterologously expressed in *E. coli* BL21. Sodium dodecyl sulfate-polyacrylamide gel electrophoresis (SDS-PAGE) analysis revealed a soluble protein of approximately 43 kDa, which corresponded to the predicted molecular mass of the recombinant protein, with the majority of the protein recovered in the soluble supernatant fraction (Figure 6A). Affinity purification yielded a single eluted band with 50 mM imidazole, confirming the isolation of homogeneous SiFRK4 protein (Figure 6B). Using the purified enzyme, an in vitro activity assay demonstrated that SiFRK4 catalyzed the phosphorylation of fructose, confirming its fructokinase function (Figure 6C). Collectively, these results established SiFRK4 as a functional fructokinase in vitro.

### 2.6. SiFRK4 Interacts with SiPhyC

**Figure 7:**
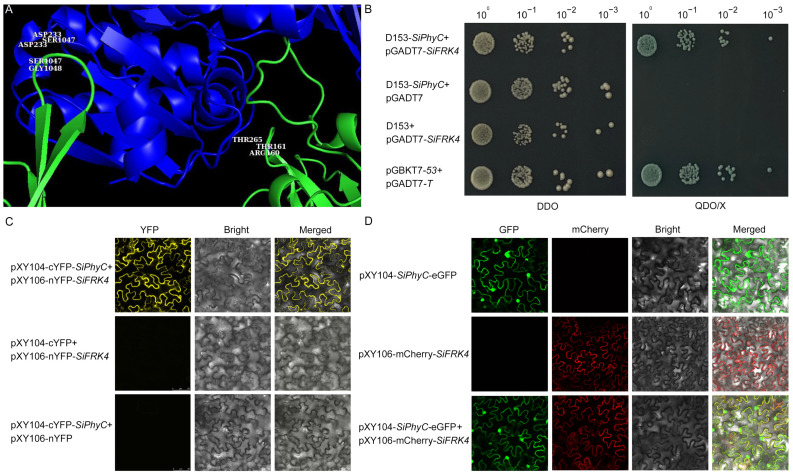
Prediction and validation of the interaction between SiFRK4 and SiPhyC proteins. (**A**) Predicted structural model of the SiFRK4-SiPhyC complex generated by AlphaFold3 and visualized with PyMOL v2.1.0. (**B**) Validation of SiFRK4-SiPhyC interaction using Y2H assay. The D153-*SiPhyC* and pGADT7-*SiFRK4* were co-transformed into the yeast strain AH109. Transformants were spotted in 10-fold serial dilutions onto DDO medium (SD/-Trp-Leu) for transformation control and QDO/X medium (SD/-Ade/-His/-Leu/-Trp with X-a-Gal) for interaction screening. Growth of blue colonies on QDO/X-α-gal medium indicated positive interaction. The pair pGBKT7-53 + pGADT7-T was used as positive control, while empty vectors served as negative control (−). (**C**) In vivo SiFRK4-SiPhyC interaction confirmed by BiFC assay. (**D**) Subcellular co-localization analysis of SiPhyC and SiFRK4 in tobacco leaves.

Given that *SiFRK4* expression exhibited a diurnal rhythm, we investigated its potential functional connection with SiPhyC, a key photoreceptor in foxtail millet [19,36]. Initial SiFRK4-SiPhyC interaction analysis using AlphaFold predicted multiple interaction interfaces between the two proteins with medium-to-high confidence (interface pTM = 0.61) (Figure 7A). To experimentally validate this prediction, we performed yeast two-hybrid (Y2H) and bimolecular fluorescence complementation (BiFC) assays. Both methods confirmed a direct physical interaction between SiFRK4 and SiPhyC in vivo (Figure 7B,C). Further characterization of their subcellular localization in *Nicotiana Benthamiana* leaves via confocal microscopy revealed that while SiPhyC was present in both the nucleus and cytoplasm, SiFRK4 was predominantly cytoplasmic. Notably, the two proteins co-localized specifically within the cytoplasmic compartment (Figure 7D), corroborating the interaction observed in the Y2H and BiFC assays.

### 2.7. SiPhyC Regulates Central Carbon Metabolism

**Figure 8:**
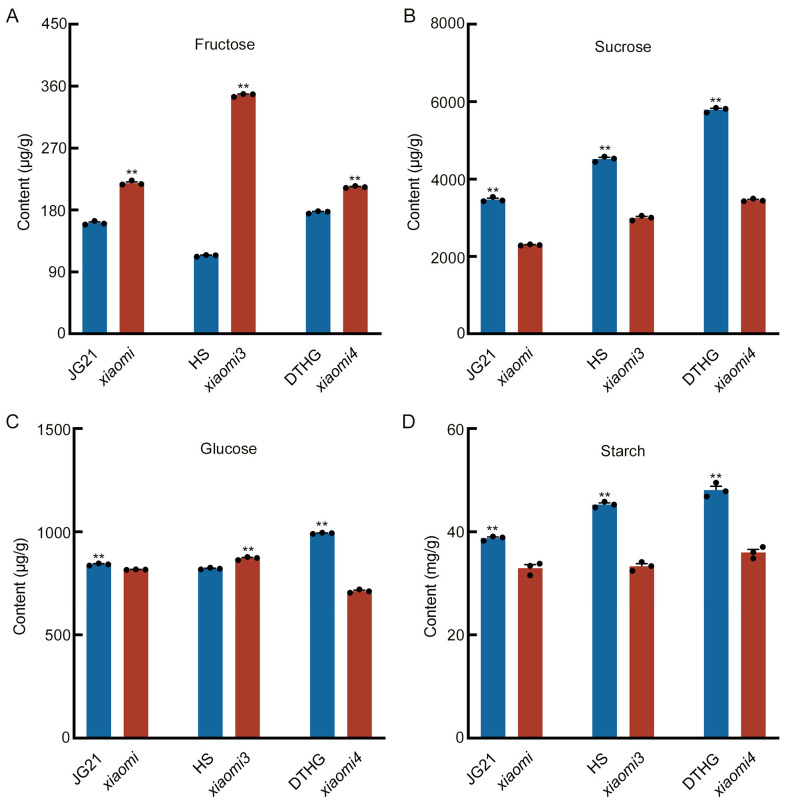
Metabolic profiling of soluble sugars and starch in *SiPhyC* mutants. (**A**–**C**) Soluble sugar contents in 14-day-old seedlings of three *SiPhyC* allelic mutants (*xiaomi*, *xiaomi3*, *xiaomi4*) and their corresponding wild-type (WT) lines: (**A**) fructose, (**B**) sucrose, (**C**) glucose. (**D**) Starch content in the same set of seedlings. Data are presented as mean ± S.D. from three independent experiments (*n* = 3). Statistical significance was determined by one-way ANOVA followed by LSD’s significant difference test. ** *p* < 0.01.

Having established the SiFRK4-SiPhyC interaction, we examined its physiological relevance by analyzing carbohydrate metabolism in three independent *SiPhyC* allelic mutants (*xiaomi*, *xiaomi3*, *xiaomi4*) and their corresponding wild-type lines (JG21, HS, DTHG). Metabolite profiling revealed profound and consistent alterations. Fructose levels were markedly elevated in all mutants compared to their respective wild-types (Figure 8A). Conversely, sucrose and starch contents were significantly reduced across all mutant backgrounds (Figure 8B,D). Glucose levels showed a more variable response, decreasing in *xiaomi* and *xiaomi4* but increasing in *xiaomi3* (Figure 8C). These data demonstrated that loss of *SiPhyC* function disrupted carbohydrate homeostasis, leading to a specific metabolic profile characterized by fructose accumulation and depletion of sucrose and starch. Together with the observed protein–protein interaction, these results suggested that SiPhyC might influence central carbon metabolism, at least in part, through its association with SiFRK4.

## 3. Discussion

Our pan-genome analysis across diverse foxtail millet accessions revealed a complex and dynamic landscape for the *SiFRK* gene family. The identification of 697 members underscores substantial genomic plasticity, with copy number per accession ranging from 5 to 9. This variation aligns with observations in other plant gene families where pan-genome analyses uncover a rich “dispensable genome” contributing to phenotypic diversity and adaptation. The PAV patterns are particularly instructive. The universal conservation of *SiFRK2* suggests a non-redundant, fundamental role, likely related to a core metabolic function indispensable across all ecotypes. Conversely, the restricted distribution of members like *SiFRK5*, *SiFRK6*, and *SiFRK8*—absent in the majority of accessions—implies they may represent lineage-specific innovations or genes under relaxed selection, possibly involved in niche adaptation. The prevalence of *SiFRK4* across wild relatives and cultivars, coupled with its significant natural polymorphism, positions it as a key evolutionary node, potentially balancing conserved enzymatic function with adaptive regulatory variation. These patterns mirror the evolutionary trajectories of metabolic gene families, where some members are tightly conserved, while others diversify, providing genetic raw material for environmental adaptation and domestication.

Our multi-faceted characterization establishes *SiFRK4* as a central player in foxtail millet biology. Its consistent high expression across developmental stages and tissues, except for specific low points in leaves, suggests a housekeeping role in fructose metabolism that is dynamically regulated rather than constitutively active (Figure 4). The pronounced expression peak in stem at booting and panicle at heading strongly implicates *SiFRK4* in the carbohydrate dynamics underpinning reproductive development and grain filling, a critical phase for yield (Figure 4B). Furthermore, the diurnal oscillation of *SiFRK4* expression, peaking at the end of the light period, integrates it into the circadian network that synchronizes metabolism with photoperiod. This rhythmicity is a hallmark of genes involved in carbon processing, ensuring metabolic efficiency by anticipating predictable environmental cycles. The rich haplotype diversity of *SiFRK4* in natural populations provides a valuable reservoir of allelic variation for dissecting genotype-phenotype relationships and for breeding programs aiming to optimize sugar metabolism for improved yield or stress resilience (Figure 5).

The most significant finding of this study is the direct physical interaction between the metabolic enzyme SiFRK4 and the phytochrome photoreceptor SiPhyC, demonstrated through complementary Y2H, BiFC, and co-localization assays (Figure 7). This discovery builds upon the known link between light signaling and carbohydrate metabolism but identifies a specific, direct protein–protein interaction as a potential mechanistic conduit. Phytochromes are well-established master regulators of plant development and physiology, perceiving red/far-red light and modulating processes from seed germination to flowering [19,36]. Their role in regulating starch and sugar metabolism has been documented, often mediated through transcriptional networks [37]. Our finding that SiPhyC interacts with a fructokinase in the cytoplasm suggests an additional, more rapid, post-translational layer of regulation. The cytoplasmic co-localization of SiFRK4 and SiPhyC is pivotal, as it positions their interaction within a major compartment of sugar metabolism, potentially allowing light signals to directly fine-tune fructose phosphorylation flux. This interaction model—a photoreceptor directly engaging a metabolic enzyme—offers a parsimonious mechanism for the immediate adjustment of carbon flow in response to light quality and quantity, complementing slower transcriptional responses.

The physiological relevance of the SiFRK4-SiPhyC interaction is compellingly supported by the metabolic phenotype of *SiPhyC* mutants (Figure 8). The consistent profile—dramatic fructose accumulation coupled with severe depletion of sucrose and starch—indicates that SiPhyC plays a critical role in carbon partitioning. The elevated fructose likely results from a bottleneck in its phosphorylation, a process where SiFRK4 is a key catalyst. One plausible hypothesis is that the SiFRK4-SiPhyC interaction modulates SiFRK4’s activity, localization, or stability; consequently, loss of SiPhyC could impair SiFRK4 function, leading to fructose build-up. The consequent reduction in sucrose and starch may reflect a downstream shortage of phosphorylated hexose precursors for their biosynthesis. The observed variation in glucose levels—decreased in *xiaomi* and *xiaomi4* but increased in *xiaomi3*—highlights the metabolic heterogeneity among allelic mutants. This inconsistency is not unexpected given that glucose is a central metabolic hub whose steady-state levels are determined by the net balance of multiple converging pathways, including sucrose hydrolysis, glucose phosphorylation by various hexokinase isoforms, and downstream glycolytic flux. The divergent glucose phenotypes may reflect allele-specific compensatory responses within this highly networked system or subtle baseline differences between the wild-type backgrounds.

The extensive haplotype diversity observed at the *SiFRK4* locus (Figure 5; Supplemental Data S3) suggests that this gene has been subject to selection during foxtail millet evolution and domestication. Regulatory and coding-sequence polymorphisms may fine-tune fructose metabolism in response to environmental variation, contributing to ecological adaptation and yield stability.

From an agronomic perspective, the interplay between SiPhyC and SiFRK4 (Figure 7 and Figure 8) represents a promising leverage point for future research aimed at optimizing carbon-use efficiency in C_4_ cereals. A deeper understanding of how this interaction affects enzyme function could eventually inform strategies to improve sugar utilization and starch accumulation without perturbing essential metabolic processes. Given the central role of carbohydrate partitioning in yield formation and stress tolerance, these findings offer new opportunities for molecular breeding and metabolic engineering.

However, this study has several limitations that warrant further investigation. Although we have established a clear physical interaction between SiPhyC and SiFRK4, the precise molecular mechanism through which SiPhyC modulates SiFRK4 function remains to be fully characterized. At present, direct evidence for *SiPhyC*-mediated changes in SiFRK4 enzyme activity or protein dynamics is lacking. Future studies should aim to detect alterations in SiFRK4 enzyme activity, protein abundance, or subcellular localization in *SiPhyC* mutant backgrounds. Addressing these aspects will be essential to determine whether SiPhyC functions as a modulator of SiFRK4 catalytic efficiency, a scaffold for its subcellular localization, or a regulator of its protein stability. Moreover, the generation of *SiFRK4* overexpression or knockout mutants will be necessary to directly confirm that *SiPhyC*-mediated regulation of carbon metabolism is dependent on *SiFRK4*.

## 4. Materials and Methods

### 4.1. Pan-Genome Identification and Sequence Analysis of the SiFRK Genes in Foxtail Millet

The pan-genome data of 110 foxtail millet accessions (including 35 cultivars, 40 landraces, and 35 wild relatives) and the T2T reference genome of the cultivar Yugu1 were retrieved from the SetariaDB “http://111.203.21.71:8000/index.html (accessed on 4 February 2026)” [38], a dedicated multi-omics database for foxtail millet.

Using the nine previously reported *FRK* genes from Yugu1 as queries, we performed homology searches across the 110 pan-genome assemblies. Candidate genes were retained if they met the following criteria: E-value = 0 and sequence coverage ≥80%. The presence of conserved FRK domains in the corresponding protein sequences was further verified using the SMART database “http://smart.embl-heidelberg.de/ (accessed on 4 February 2026)”. PAV of *SiFRK* genes across accessions was visualized as a heatmap using the GenesCloud platform “https://www.genescloud.cn (accessed on 4 February 2026)”.

Protein sequences of all identified SiFRK members were aligned using MUSCLE, and a maximum-likelihood phylogenetic tree was constructed with IQ-TREE2. The substitution model was automatically selected by ModelFinder (MFP), and branch support was assessed with 1000 ultrafast bootstrap replicates. Conserved protein motifs were predicted using the MEME online tool “https://meme-suite.org/meme/tools/meme (accessed on 4 February 2026)”, and conserved domains were annotated via the NCBI Conserved Domain Database “https://www.ncbi.nlm.nih.gov/Structure/bwrpsb/bwrpsb.cgi (accessed on 4 February 2026)”. Promoter sequences (2 kb upstream of the translation start site) for the nine *SiFRK* genes were retrieved from the Yugu1 genome and analyzed for *cis*-acting regulatory elements using the PlantCARE database “http://bioinformatics.psb.ugent.be/webtools/plantcare/html/ (accessed on 4 February 2026)”. All resulting data were visualized using TBtools-II [39].

### 4.2. Expression Pattern Analysis

Public transcriptome profiles for 28 tissues across seven developmental stages of the foxtail millet cultivar Yugu1 were obtained from SetariaDB [38]. Expression patterns of the nine *SiFRK* genes were visualized as a heatmap using the GenesCloud platform “https://www.genescloud.cn/chart/HeatMap (accessed on 4 February 2026)”, based on FPKM values.

For qRT-PCR analysis, total RNA was extracted from various tissues of the foxtail millet cultivar JG21 using the RNAprep Pure Plant Kit (Tiangen Biotech CO., LTD, Beijing, China, Cat#DP432), and cDNA was synthesized using the PrimeScript RT Reagent Kit with gDNA Eraser (Takara Biomedical Technology Co., Ltd., Beijing, China, Cat# RR047A). Quantitative RT-PCR was performed using TB Green^®^ Premix Ex Taq^™^ II (Takara Biomedical Technology Co., Ltd., Beijing, China, Cat#RR820A) on a Bio-Rad CFX96^TM^ Touch system (Bio-Rad Laboratories, Hercules, CA, USA). *SiH3.3* was used as the internal reference gene. Data were analyzed using the 2^−ΔΔCT^ method with three biological replicates. Statistical analysis was performed using DPS v7.05, and graphs were generated with GraphPad Prism 8 software.

### 4.3. Prokaryotic Expression, Purification, and Enzymatic Activity Analysis of the Fructokinase SiFRK4

The CDS of *SiFRK4* was cloned into the pET28a(+) vector to generate the recombinant plasmid pET28a(+)-*SiFRK4*. This construct was transformed into *E. coli* BL21 strain, with the empty pET28a(+) vector serving as a negative control. Recombinant protein expression was induced by adding 1 mM IPTG at 16 °C, and purified using High Affinity Ni-NTA Resin (Genscript Biotechnology Co., Ltd., Nanjing, China, Cat# L00250) according to the manufacturer’s protocol. Protein purity and molecular weight were assessed by SDS-PAGE. The fructokinase activity was measured using the Fructokinase Assay kit (Jonlnbio Co., Ltd., Shanghai, China, Cat#JL-T2656) according to the manufacturer’s instructions. Briefly, 20 µL of purified protein solution (with protein concentration normalized to 0.3 mg/mL across all samples) was mixed with the kit’s reagents 2, 3, and 4, incubated at 37 °C for 5 min, followed by addition of reagent 5 to initiate the reaction in a 96-well plate. The increase in absorbance at 340 nm was monitored at 37 °C for 20 min. Each sample was assayed in triplicate. Fructokinase activity was calculated based on the liquid volume method and expressed as nmol/min/mL.

### 4.4. Protein–Protein Interaction Prediction

Protein–protein interaction predictions were performed using the AlphaFold server "https://alphafoldserver.com/ (accessed on 4 February 2026)”. Structural models were visualized and analyzed in PyMOL v2.1.0, where amino acid residues located within 2.3 Å of potential binding interfaces were identified and mapped for further evaluation.

### 4.5. Yeast Two-Hybrid Assay

Protein–protein interaction between SiFRK4 and SiPhyC was examined using the Y2H system. The CDSs of *SiFRK4* and *SiPhyC* were cloned into the prey plasmid pGADT7 and bait vector D153, respectively. Following sequence verification, the two recombinant plasmids were co-transformed into the *Saccharomyces cerevisiae* strain AH109 using the PEG/LiAc yeast transformation protocol (Clontech, Mountain View, CA, USA). Positive transformants were selected on SD/–Trp/–Leu medium and further confirmed by yeast colony PCR. To assess interaction, yeast cultures were serially diluted (10-fold steps) and spotted onto two types of selective media: DDO and QDO/X. Plates were incubated at 30 °C for 3–5 days, and growth as well as blue colony development were recorded as evidence of protein interaction.

### 4.6. The Bimolecular Fluorescence Complementation Assay

To examine the potential interaction between SiFRK4 and SiPhyC in planta, a BiFC assay was performed. The CDSs of *SiPhyC* and *SiFRK4* were cloned into the pXY104-cYFP and pXY106-nYFP vectors, respectively. After verification by sequencing, the recombinant constructs were introduced into *Agrobacterium tumefaciens* strain GV3101. Bacterial cultures were grown to log phase, harvested, and resuspended in infiltration buffer (10 mM MES, 10 mM MgCl_2_, 200 µM acetosyringone) to an optical density at 600 nm (OD_600_) of 1.0 for each strain. Equal volumes of the two bacterial suspensions were mixed and infiltrated into the abaxial side of young leaves of *Nicotiana benthamiana* plants using a needle-free syringe. Following infiltration, plants were kept in darkness for 12 h and then transferred to normal growth conditions for an additional 48 h. Reconstituted YFP fluorescence was visualized using a confocal laser-scanning microscope (Leica TCS SP8, Leica Microsystems, Wetzlar, Germany). All experiments were performed in three independent biological replicates, with at least three plants per replicate and three leaves inoculated per plant.

### 4.7. Subcellular Co-Localization Analysis of SiFRK4 and SiPhyC via Transient Expression

To investigate the subcellular interaction between SiFRK4 and SiPhyC, co-localization assays were performed using fluorescence-tagged constructs in *Nicotiana benthamiana* leaves. The CDSs of *SiFRK4* and *SiPhyC* were cloned in-frame into the enhanced green fluorescent protein (eGFP) in vector pXY104 and the mCherry fluorescent protein in vector pXY106, respectively. For transient co-expression, bacterial suspensions carrying each construct were adjusted to an optical density at 600 nm (OD600) of 0.6–0.8, mixed at a 1:1 volume ratio, and co-infiltrated into leaves of *Nicotiana benthamiana* plants. After incubation for 48–72 h, fluorescence was observed by confocal laser scanning microscope.

### 4.8. Measurements of Starch and Soluble Sugar Content in Foxtail Millet Seedlings

Fourteen-day-old seedlings of *SiPhyC* allelic mutants (*xiaomi*, *xiaomi3* and *xiaomi4*) and wild types (JG21, HS and DTHG) were collected one hour before the end of the light period and immediately frozen in liquid nitrogen. For soluble sugar extraction, including fructose, glucose, and sucrose, 100 mg of freeze-dried powder was homogenized with 80% (*v*/*v*) acetonitrile. The mixture was ultrasonicated at 4 °C for 30 min and centrifuged at 5000× *g* for 10 min. The extraction procedure was repeated once, and the combined supernatants were filtered through a 0.22 μm membrane and stored at 4 °C until further analysis. Soluble sugars were separated using an Agilent 1260 HPLC system (Agilent Technologies, Inc., Santa Clara, CA, USA) equipped with an evaporative light scattering detector (ELSD). Separation was achieved on a Waters XBridge Amide column (4.6 × 150 mm, 5 μm). The mobile phase consisted of solvent A (30% acetonitrile with 0.2% triethylamine) and solvent B (80% acetonitrile with 0.2% triethylamine), with the following gradient elution program: 0–10 min, 100% B; 10–23 min, 100–50% B; 23–23.1 min, 50–100% B; 23.1–26 min, 100% B. The flow rate was 1.0 mL·min^−1^, the column temperature was maintained at 35 °C, and the injection volume was 10 μL. External calibration was performed using pure sugar standards in the concentration range of 12.5–800 μg·mL^−1^.

Starch content was determined using the acid hydrolysis–DNS method. Lipids and soluble sugars were first removed from the samples using petroleum ether and 85% ethanol. The resulting pellet was then hydrolyzed with 3 mL of HCl (1:1, *v*/*v*) under reflux in a boiling water bath for 2 h. After hydrolysis, the solution was neutralized, clarified with lead acetate and sodium sulfate, and filtered. The glucose released from starch hydrolysis was quantified colorimetrically at 540 nm using the DNS reagent. A standard curve was prepared using glucose solutions ranging from 0 to 1 mg/mL. Starch content was calculated as glucose equivalents multiplied by a conversion factor of 0.9 and expressed per gram of dry weight. All measurements were performed with three biological replicates. Statistical analysis was performed using one-way ANOVA followed by LSD’s significant difference test in DPS software version 7.5.

## 5. Conclusions

In conclusion, this study reveals that fructokinase function in a C_4_ cereal is not merely constitutive but can be linked to environmental signaling. Through pan-genome analysis (Figure 1 and Figure 2), expression profiling (Figure 4), biochemical validation (Figure 6), protein interaction assays (Figure 7), and mutant-based metabolic analyses (Figure 8), we identify SiFRK4 as a conserved, rhythmically regulated fructokinase that physically interacts with the photoreceptor SiPhyC. This interaction may represent a previously unrecognized light-metabolism regulatory axis influencing carbon partitioning in foxtail millet.

## Figures and Tables

**Table 1:** Gene and protein features of identified SiFRKs in Yugu1.

Gene Name	Gene ID	CDS Length (bp)	Protein Length (aa)	Molecular wt. (kDa)	PI	Gravy
*SiFRK1*	*Seita.4G101200*	1170	389	41.11	5.87	0.050
*SiFRK2*	*Seita.5G383200*	1587	528	59.67	6.24	−0.630
*SiFRK3*	*Seita.5G411800*	972	323	34.65	5.05	0.154
*SiFRK4*	*Seita.6G090000*	1005	334	35.40	5.34	0.092
*SiFRK5*	*Seita.6G203900*	1215	404	42.90	6.04	0.013
*SiFRK6*	*Seita.6G254800*	1092	363	38.54	5.03	0.062
*SiFRK7*	*Seita.7G320600*	1098	365	38.76	4.85	0.052
*SiFRK8*	*Seita.9G173300*	1746	581	64.90	7.56	−0.544
*SiFRK9*	*Seita.9G229500*	1224	407	43.03	5.84	0.128

## Data Availability

The original contributions presented in this study are included in the article/Supplementary Materials. Further inquiries can be directed to the corresponding author.

## Supplementary Materials

The following supporting information can be downloaded at: https://www.mdpi.com/article/10.3390/plants15060907/s1. Supplementary Data S1. Gene ID of the 697 *SiFRKs*. Supplementary Data S2. The CDSs of the 697 *SiFRKs*. Supplementary Data S3. Haplotypes of the *SiFRK* genes. Figure S1. Motif distributions and gene structures of *SiFRK1* in 109 pangenome accessions. Figure S2. Motif distributions and gene structures of *SiFRK2* in 110 pangenome accessions. Figure S3. Motif distributions and gene structures of *SiFRK3* in 108 pangenome accessions. Figure S4. Motif distributions and gene structures of *SiFRK4* across 109 pangenome accessions. Figure S5. Motif distributions and gene structures of *SiFRK5* in 3 pangenome accessions. Figure S6. Motif distributions and gene structures of *SiFRK6* in 3 pangenome accessions. Figure S7. Motif distributions and gene structures of *SiFRK7* in 99 pangenome accessions. Figure S8. Motif distributions and gene structures of *SiFRK8* across in pangenome accessions. Figure S9. Motif distributions and gene structures of *SiFRK9* across in pangenome accessions.
