# Supplementary material for: Pan-Genome Analysis of the Fructokinase Gene Family Reveals a Light-Regulated SiPhyC–SiFRK4 Module Controlling Carbon Partitioning in Foxtail Millet
Source: Plants (Basel). 2026 Mar 15;15(6):907. doi: 10.3390/plants15060907 (PMC13030009; doi:10.3390/plants15060907)
Supplement: Supplementary file 1 [file plants-15-00907-s001.zip › Figure S1-S9.pdf]

Supplementary Figures

Pan-Genome Analysis of the Fructokinase Gene Family Reveals a Light-Regulated SiPhyC–SiFRK4 Module Controlling Carbon Partitioning in Foxtail Millet

Lu He [1,2], Juan Zhao [3], Guangxin Wang [3], Ling Yuan [4], Xingchun Wang [1,5] and Zhirong Yang [1,3]*

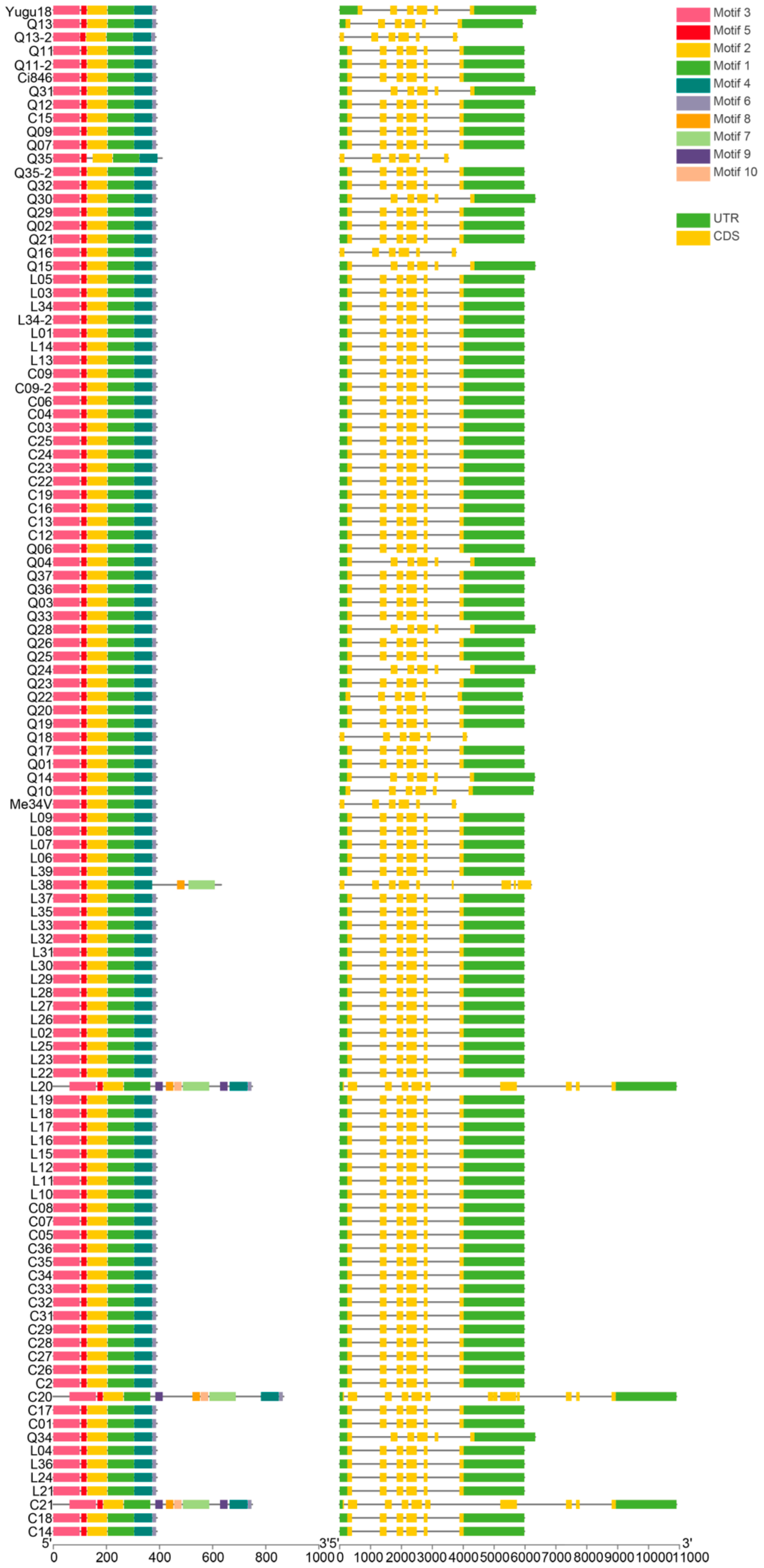

Figure S1. Motif distributions and gene structures of *SiFRK1* in 109 pangenome accessions.

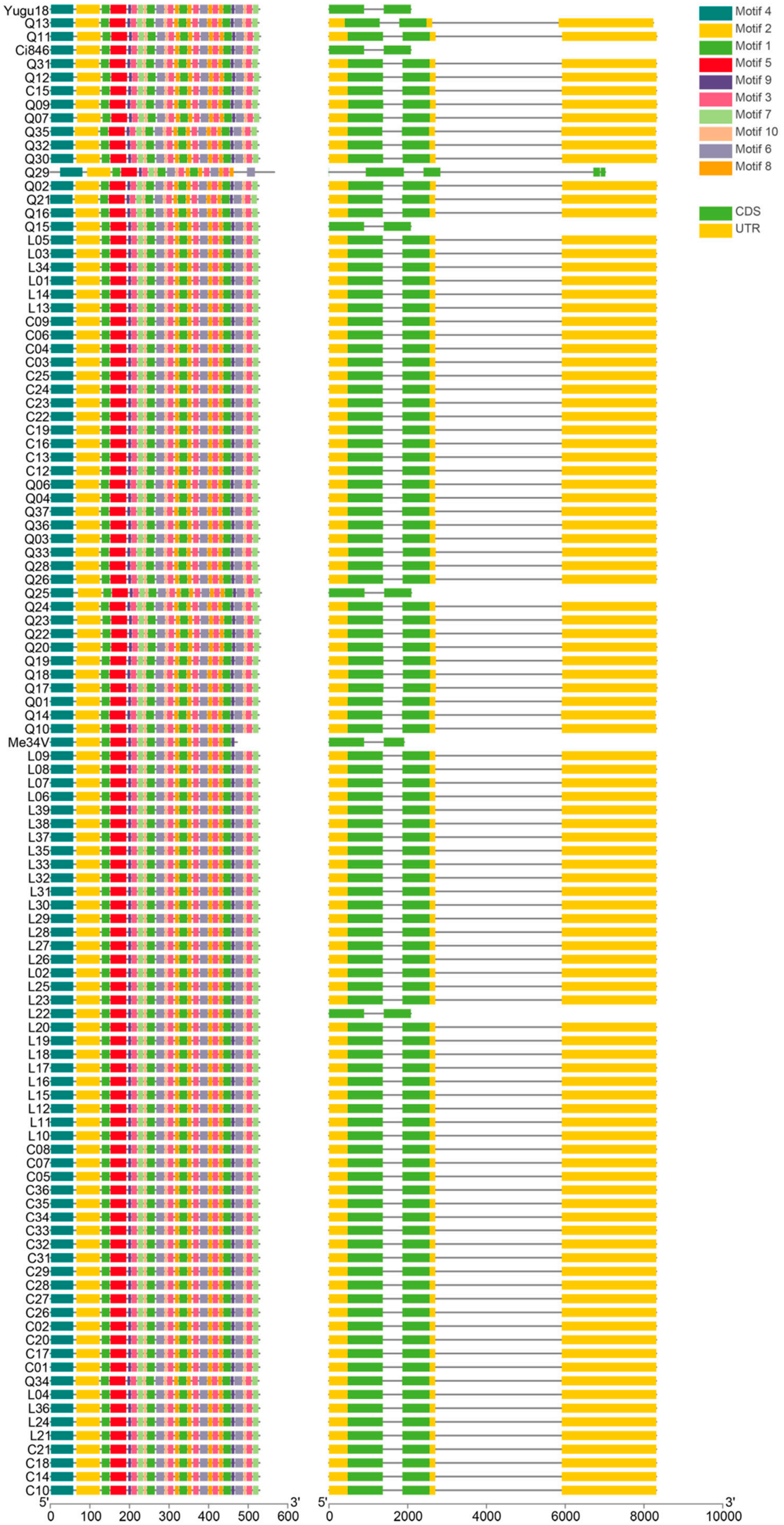

Figure S2. Motif distributions and gene structures of *SiFRK2* in 110 pangenome accessions.

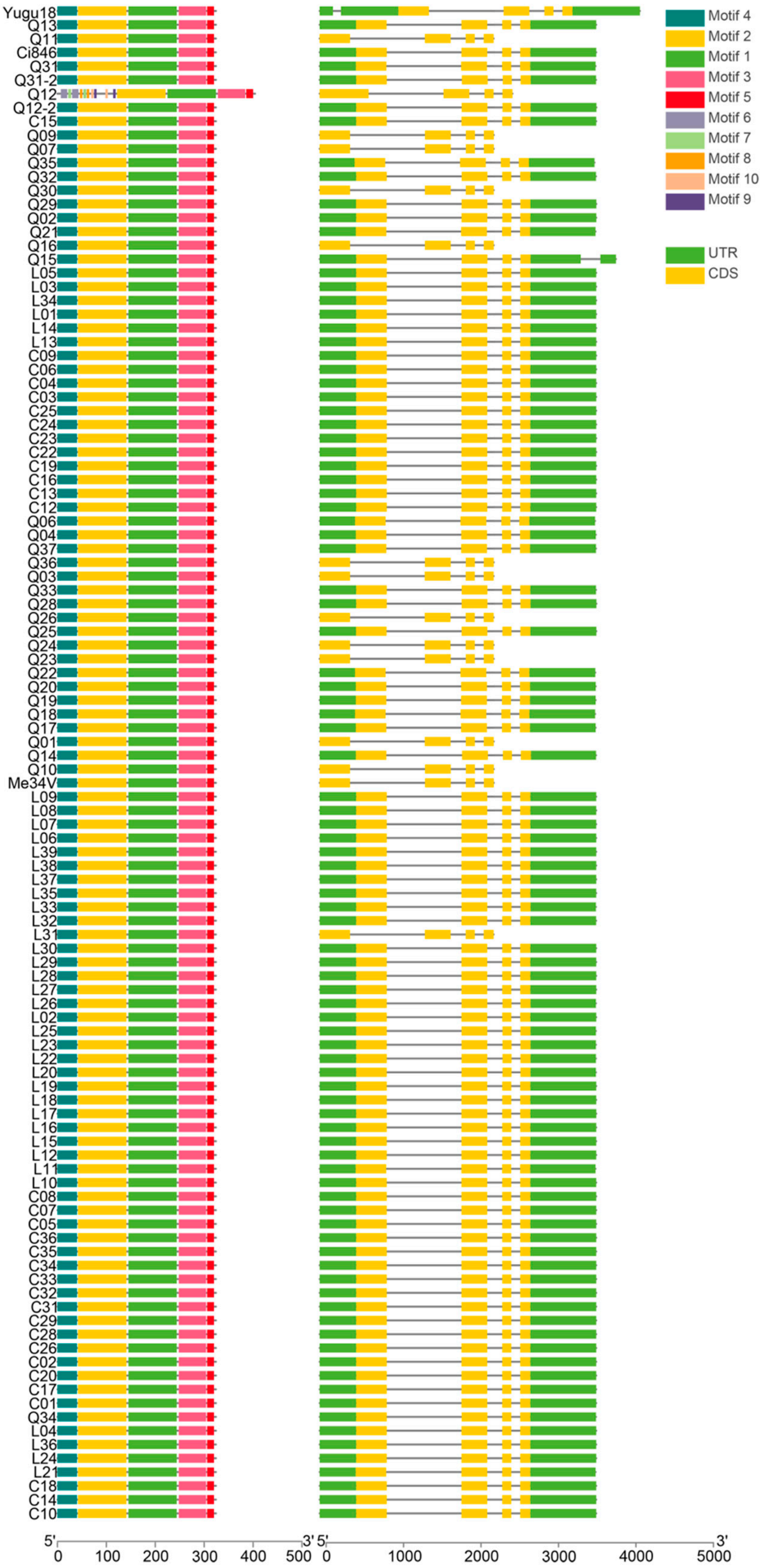

Figure S3. Motif distributions and gene structures of *SiFRK3* in 108 pangenome accessions.

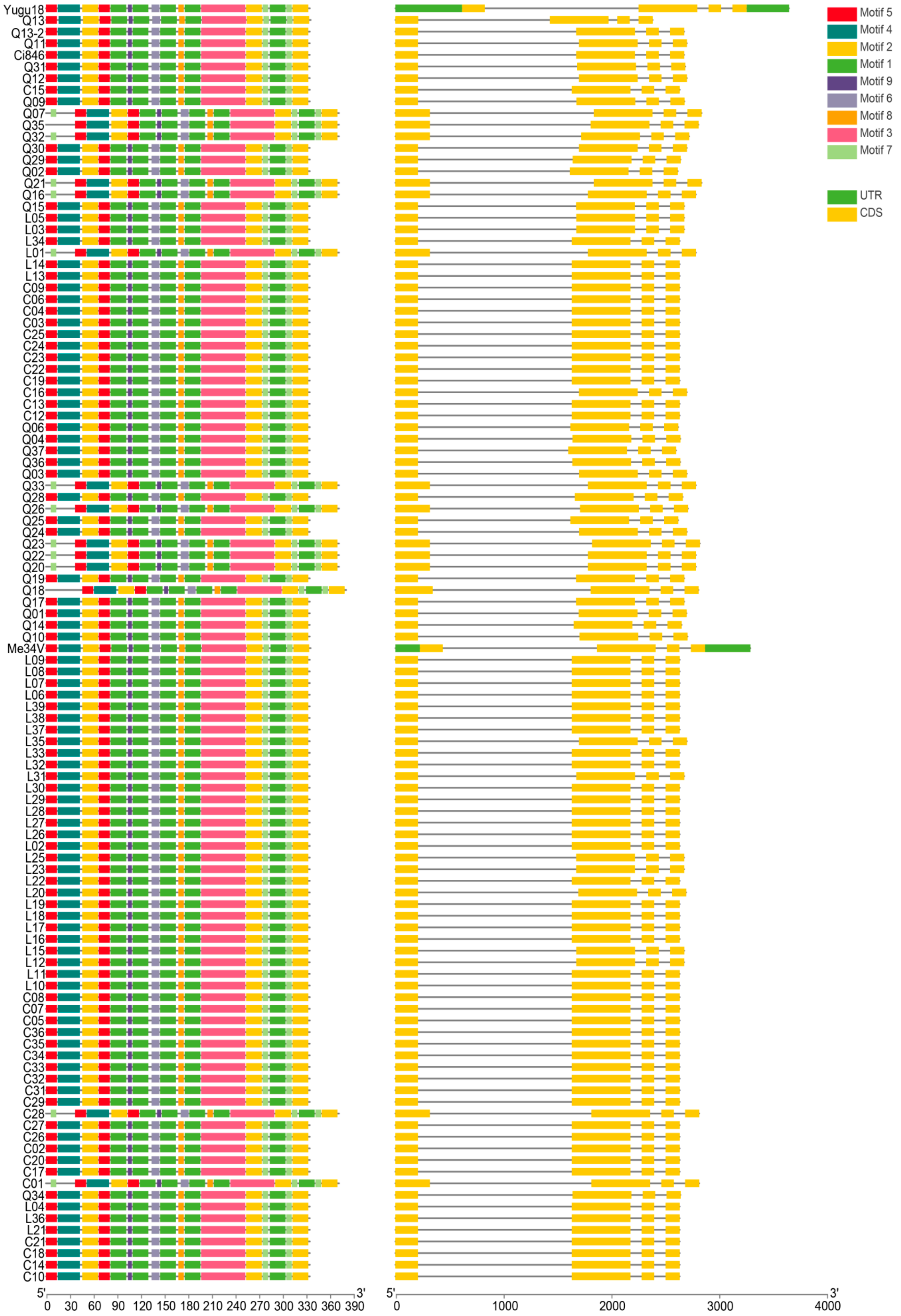

Figure S4. Motif distributions and gene structures of *SiFRK4* across 109 pangenome accessions.

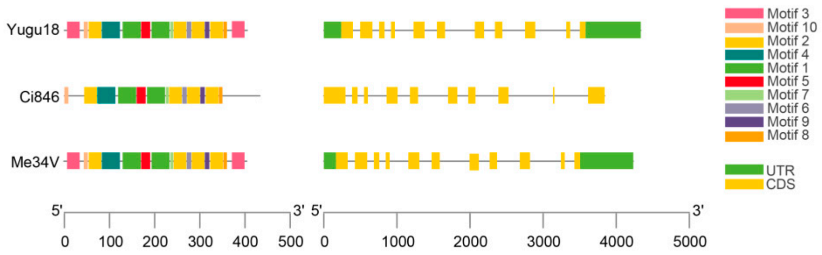

Figure S5. Motif distributions and gene structures of *SiFRK5* in 3 pangenome accessions.

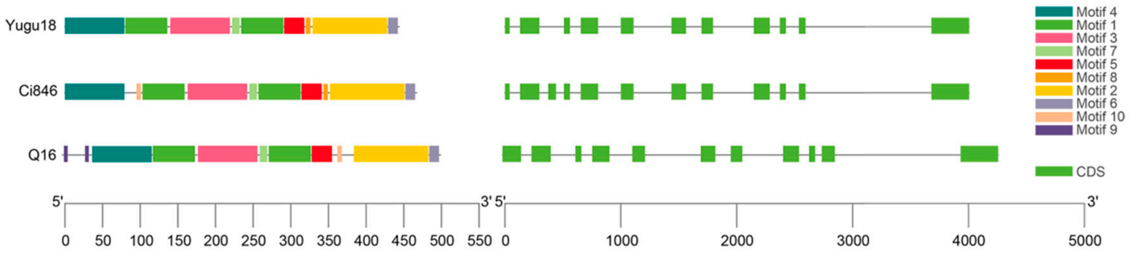

Figure S6. Motif distributions and gene structures of *SiFRK6* in 3 pangenome accessions.

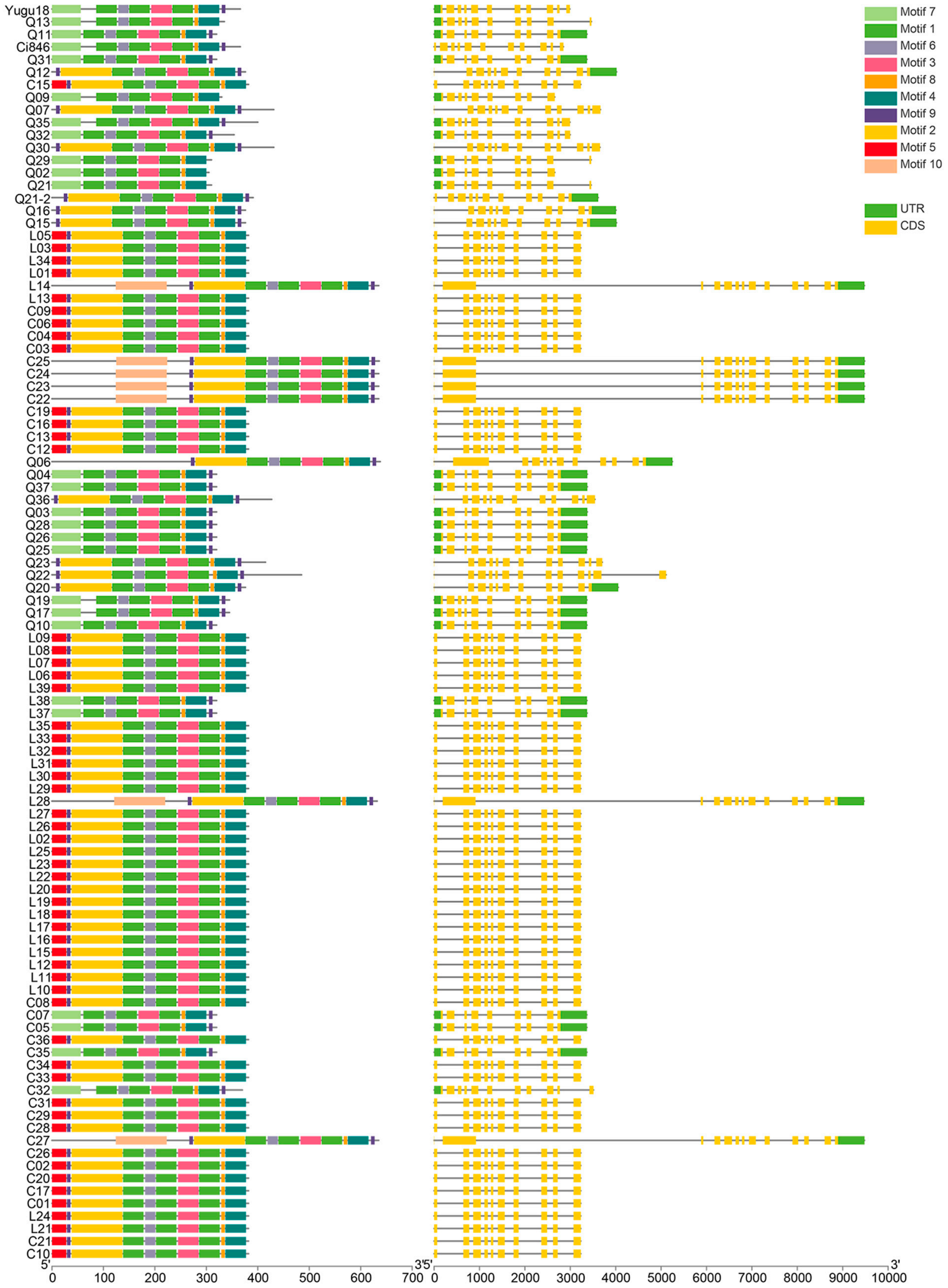

Figure S7. Motif distributions and gene structures of *SiFRK7* in 99 pangenome accessions.

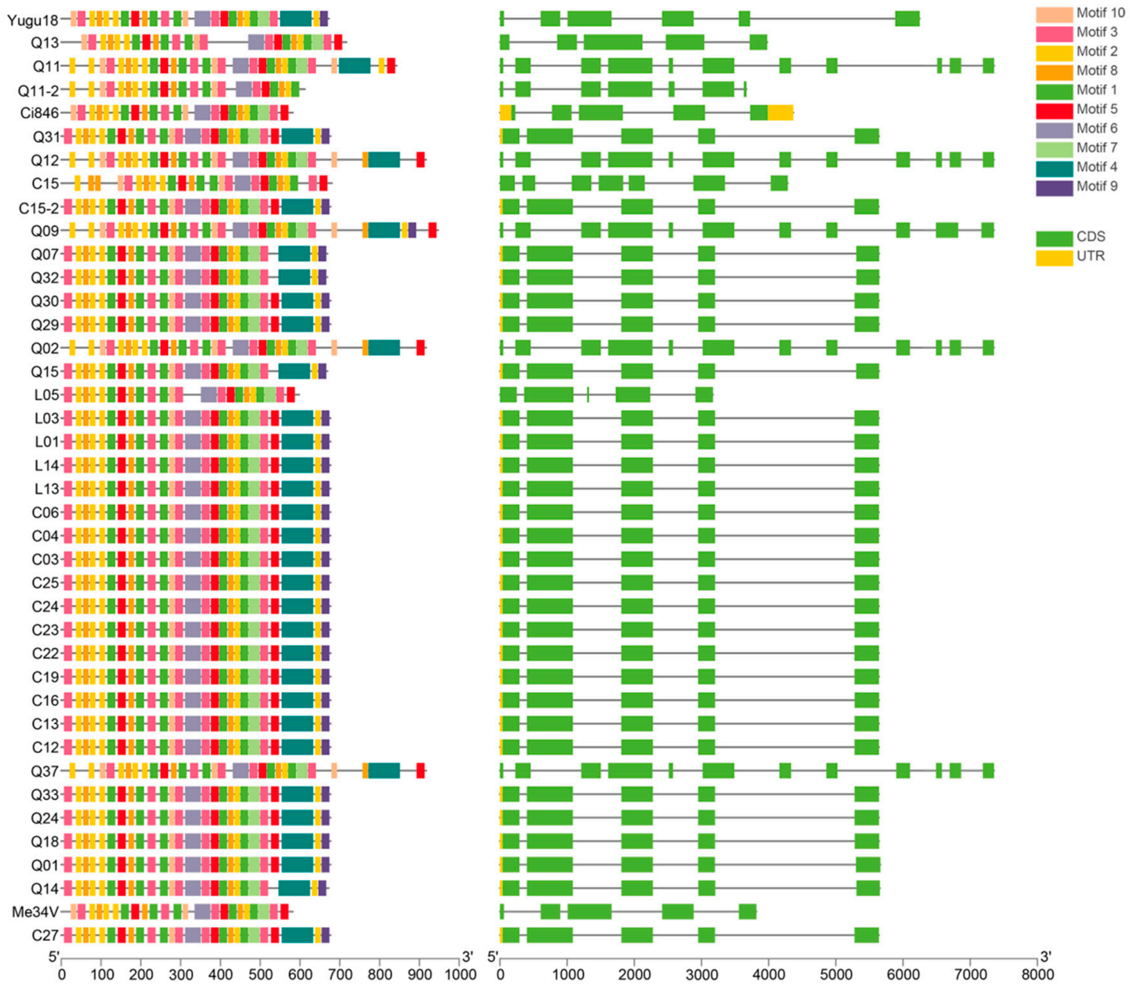

Figure 8. Motif distributions and gene structures of *SiFRK8* across in 38 pangenome accessions.

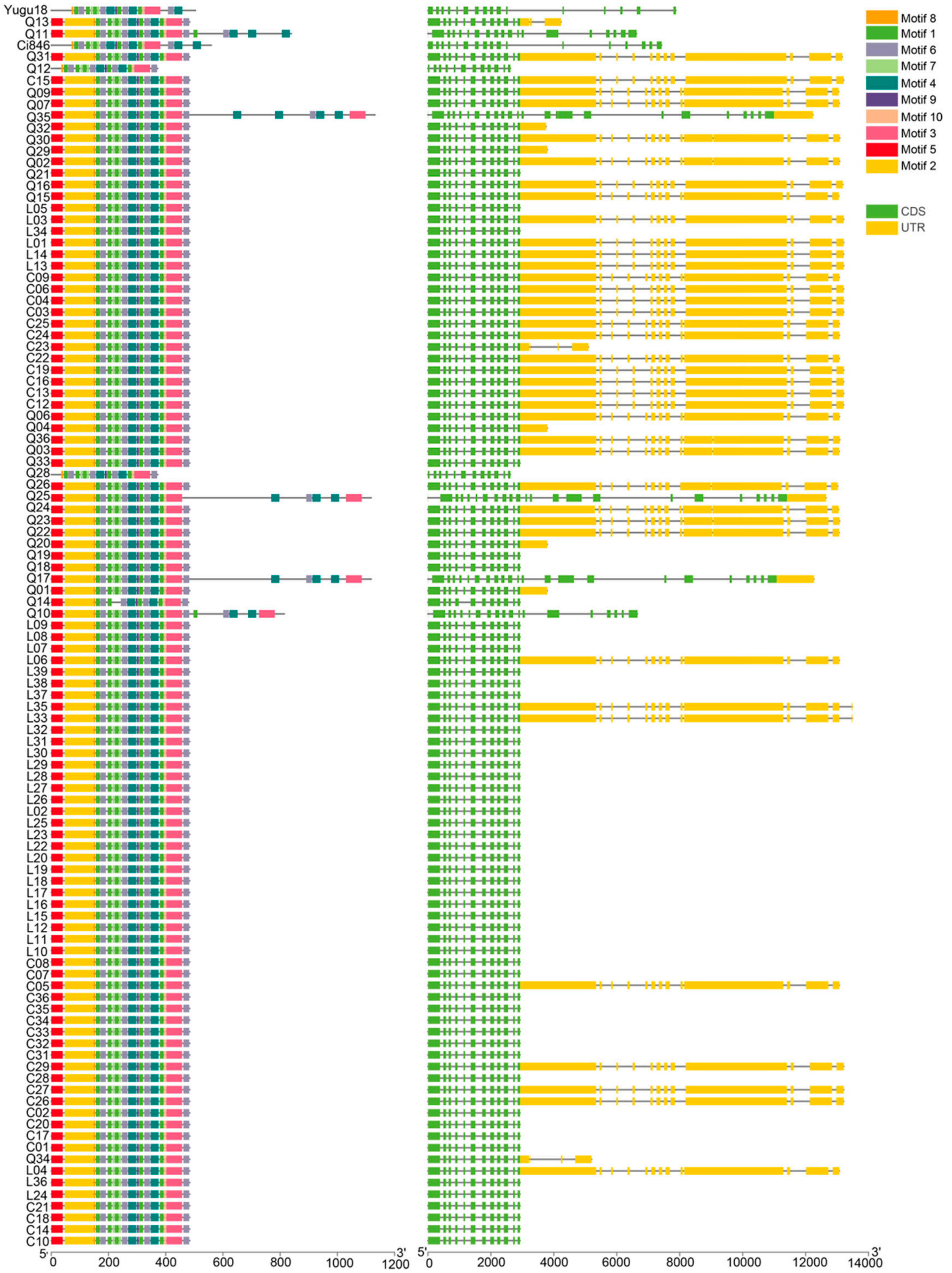

Figure S9. Motif distributions and gene structures of *SiFRK9* across in 107 pangenome accessions.
